# Probiotics reduce negative mood over time: the value of daily self-reports in detecting effects

**DOI:** 10.1038/s44184-025-00123-z

**Published:** 2025-04-09

**Authors:** Katerina V.-A. Johnson, Laura Steenbergen

**Affiliations:** 1 Clinical Psychology Unit, Leiden University, Institute of Psychology, Leiden, The Netherlands; 2 Department of Psychiatry, University of Oxford, Oxford, UK

**Keywords:** Emotion, Psychology, Microbiome

## Abstract

The burgeoning field of the microbiome–gut–brain axis has inspired research into how the gut microbiome can affect human emotion. Probiotics offer ways to investigate microbial-based interventions but results have been mixed, with more evidence of beneficial effects in clinically depressed patients. Using a randomised, double-blind, placebo-controlled design in 88 healthy volunteers, we conduct a comprehensive study into effects of a multispecies probiotic on emotion regulation and mood through questionnaires, emotional processing tests and daily reports. We find clear evidence that probiotics reduce negative mood, starting after two weeks, based on daily monitoring, but few other changes. Our findings reconcile inconsistencies of previous studies, revealing that commonly used pre- versus post-intervention assessments cannot reliably detect probiotic-induced changes in healthy subjects’ emotional state. We conclude that probiotics can benefit mental health in the general population and identify traits of individuals who derive greatest benefit, allowing future targeting of at-risk individuals.

## Introduction

Finding ways to improve mental health and prevent the onset of clinical psychological symptoms is becoming an ever more important challenge for society. While substantial advances have been made in the fields of psychology, psychiatry and neuroscience, there remains the urgent need to identify novel and effective interventions, including pharmacotherapies^1^, neural stimulation^2^, behavioural therapies^3^ and nutritional supplements^4^.

In the past decade, there has been growing interest in understanding how the gut microbiome (the community of microorganisms living in the gut) can modulate brain function and its potential to provide new insight into the factors influencing mental health, as well as possible treatments^5^. Research on the microbiome–gut–brain axis has demonstrated that the gut microbial community can influence brain development, neurochemistry and behaviour^6–9^. The causal contribution of the gut microbiome to brain function and behaviour is most clearly demonstrated using transplantation studies in animals, whereby behavioural phenotypes can be transferred via the gut microbiota^10^. Several independent studies have now shown that rodents colonised with the gut microbiota of humans suffering from depression (but not those colonised with the gut microbiota of healthy humans) display changes characteristic of depression at both a physiological and behavioural level^11,12^.

There are clear mechanistic pathways that may mediate interactions between the gut microbiome and the brain, most notably via neural, immune, and endocrine signalling^13–16^. For example, in animals the probiotic *Lactobacillus rhamnosus* can increase stimulation of vagal afferents^17^ and was shown to reduce anxiety and depressive-like behaviours only when the vagus nerve was functionally intact^18^. Probiotic administration in animals has also been found to reduce the levels of pro-inflammatory cytokines^19^ which is relevant since immune status has been increasingly linked to neuropsychiatric conditions such as depression^20,21^. In addition, the gut microbial community interacts with the body’s hormones and probiotics have been shown to reduce corticosterone concentration in rodents^18,22^ and there is also evidence that probiotics and prebiotics can lower cortisol levels in humans^23,24^.

Since the gut microbiome interacts with these important physiological systems, it represents a possible avenue via which brain function can be indirectly modulated. With animal studies revealing that the gut microbiome can affect social behaviour, anxiety and depressive-like symptoms and responses to stress^6,25^, this indicates that the human gut microbiome may play a role in mental health and disorders. Indeed, psychological conditions are often comorbid with gastrointestinal problems^26^. Following numerous animal studies finding a beneficial effect of probiotics on the brain and behaviour, there has been much interest to determine whether such findings translate to humans. This led to many studies investigating the effects of microbiome-focused interventions on human psychology, most notably probiotics (live bacteria) and also prebiotics (selectively fermented indigestible carbohydrates such as fructo- and galacto-oligosaccharides which promote the growth and/or activity of certain gut bacteria).

A number of studies in humans have found that probiotics can improve symptoms of depression, anxiety and stress in healthy volunteers as well as clinical populations^23,27–29^. However, findings from animal studies showing preclinical efficacy of certain probiotics do not always translate to humans^30^ and there are various studies that have reported little or no effect of probiotics, motivating meta-analytical research into their effects. While probiotic studies have yielded mixed results, meta-analyses do find evidence overall of a beneficial effect on stress, anxiety and depressive symptoms^31–35^, though the weight of evidence is considerably stronger for those already suffering from psychological conditions and any benefit of probiotics for healthy populations remains unclear.

Here, we focus our investigation on assessing the effects of probiotics on emotion regulation (the ability to control one’s experience and expression of emotions) since this is a crucial and transdiagnostic factor in many mental disorders^36^. Indeed, the microbiome has been increasingly linked to human emotion^37–39^ and brain imaging has revealed that consumption of probiotics in healthy volunteers can influence neural signatures of emotion^40,41^ (though some results find this is only the case when participants are under stress^42^). It is uncertain how much any changes at the neural level resulting from probiotics translate to differences in subjective feeling. The majority of studies investigating the effects of probiotics in healthy subjects rely on questionnaires assessing symptoms of depression, anxiety or stress and often produce inconsistent results. In our study we set out to use a combination of methods to capture how probiotics influence emotion. In addition to commonly used questionnaires to assess aspects of emotion regulation, we also implemented daily monitoring to achieve temporal resolution and tests of emotional processing to detect changes at the cognitive regulatory level. By incorporating questionnaires, together with more fine-scale approaches, this study sought to provide a comprehensive understanding of the psychological effects of taking probiotics in a young and healthy adult population.

## Methods

### Participants

Sample size was determined based on a power calculation (parameters: power = 0.9, alpha = 0.05, 2 groups, 2 measurement points) in G*Power 3.1.9.7^43^. The effect size of interest was based on the smallest significant effect (*η*^2^_p_ = 0.115) of the same four-week probiotic intervention on cognitive reactivity to sad mood^29^. For the current study, the estimated sample size was 84 (42 per group) but given the possibility of drop-out, we aimed to include 90 individuals. Indeed, two participants who had been recruited as part of the probiotic group were not able to be included since the full information they provided revealed they did not comply with the exclusion criteria and one participant in the placebo group dropped out during the intervention.

Participants were eligible if they had not taken antibiotics or probiotics in the past three months, had a body mass index between 18 and 30, and did not use any medication other than hormonal contraceptives for females. Additional exclusion criteria were: consumption of more than 20 units of alcohol per week; use of soft or hard drugs more than once a month; renal, hepatic or gastrointestinal diseases or complaints; any central nervous system trauma or disorder; any past or present diagnosis of a psychological or psychiatric disorder; participation in a dietary programme or implemented structural changes in diet in the past 3 months; any hypersensitivity or allergy to nutrients such as gluten, milk, soy or peanuts.

Written informed consent was obtained from all participants before inclusion. Participants were informed they would receive either probiotics or placebo, but which intervention they received would only be disclosed after data collection was completed. This protocol and procedure conformed to the ethical standards of the 1975 Declaration of Helsinki, as revised in 1983, and were approved by the local ethics committee (Psychology Research Ethics Committee, Institute of Psychology, Leiden University, #0311194).

### Study design

A randomised, double-blind, placebo-controlled design was used to investigate the effect of 28 days of a multispecies probiotic on emotional state and emotional processing in healthy volunteers. Following the pre-intervention testing, participants assigned to the probiotics condition were provided with 30 sachets (one for each day of intervention plus two reserves) containing 2 g (2.5 × 10^9^ colony forming units (CFU) per gram) freeze-dried powder of the probiotic mixture Ecologic® Barrier (Winclove Probiotics B.V.). This mixture contains nine bacterial strains: *Bifidobacterium bifidum* W23*, B. lactis* W51 and W52*, Lactobacillus acidophilus* W37, *Levilactobacillus brevis* W63 *(*formerly classified as *Lactobacillus brevis* W63)*, Lacticaseibacillus casei* W56 (formerly classified as *Lactobacillus casei* W56), *Ligilactobacillus salivarius* W24 (formerly classified as *Lactobacillus salivarius* W24) and *Lactococcus* *lactis* W19 and W58. Participants assigned to the placebo condition were provided with 30 sachets containing 2 g freeze-dried powder of the carrier of the probiotic product: maize starch and maltodextrins. The placebo was indistinguishable from the probiotic in colour, taste and smell. Participants were instructed to dissolve these sachets in lukewarm water daily for a period of four weeks.

Afterwards, the participants underwent the post-intervention assessment, following the same procedures as the pre-intervention assessment and at the same time of day. During the four-week intervention, participants were reminded daily to take their sachet and asked to complete the daily reporting. Compliance to the intervention was assessed by counting the number of empty and full sachets returned by participants at the post-intervention session.

### Questionnaire measures

Both directly before and after the four-week intervention, participants completed a range of commonly used questionnaires measuring constructs relating to emotion regulation: the State–Trait Anxiety Inventory^44^ (STAI); the Penn State Worry Questionnaire^45^ (PSWQ); the Perceived Stress Scale^46^ (PSS); the Leiden Index of Depression Sensitivity – Revised^47^ (LEIDS-R); the Centre for Epidemiological Studies Depression Scale^48^ (CES-D); the Positive and Negative Affect Schedule^49^ (PANAS); the Emotion Reactivity Scale^50^ (ERS); the Multidimensional Assessment of Interoceptive Awareness^51^ (MAIA); the Bermond–Vorst Alexithymia Questionnaire^52^ (BVAQ) and the Buss–Perry Aggression Questionnaire^53^ (BPAQ). The participants also filled out a bowel complaints questionnaire (based on a questionnaire developed to assess functional gastrointestinal disorders^54^), with a particular focus on assessing symptoms that can be experienced by some individuals when first taking probiotics such as constipation and bloating.

### Tests of emotional processing

E-Prime 2.0 software (Psychology Software Tools, Inc., Pittsburgh, PA) was used to programme the tasks, present stimuli and collect participants’ responses. The emotional dot-probe task was used to assess selective attention for facial emotional expressions (emotional bias) and the facial expression recognition task was used to assess the accuracy in identifying emotions. These tasks have been shown to detect the early effects of pharmaceutical drugs on emotional processing in depression and have also been validated in healthy individuals^55–58^.

For the emotional dot-probe task, participants were shown two faces presented concurrently for 500 ms; a face expressing an emotion and a face with a neutral expression. Immediately after the pictures disappeared, the probe (depicted as two dots) re-appeared at one of the locations and participants were asked to click the probe as quickly as possible. The experimental task consisted of ten practice trials and 120 test trials. Test trials comprised stills of peak emotional expressions of eleven male and nine female actors from the Amsterdam Dynamic Facial Expressions Set^59^ depicting six expressions (sadness, fear, anger, happiness, surprise and neutral). Trials were counterbalanced regarding the location of the emotional picture (left or right) and location of the probe (congruent with the emotional picture or incongruent with the emotional picture). An emotional bias score was calculated for each emotion using the following formula: RT_neutralprobe_ – RT_emotionalprobe_ (where RT is reaction time). Responses faster than 200 ms or slower than 2000 ms were filtered out, as is common practice^60,61^.

For the facial expression recognition task (FERT)^62^, five basic facial expressions (happiness, anger, fear, sadness and disgust) were shown with an emotion intensity ranging from 10% to 100% in steps of 10%. Participants were first shown twelve practice trials to familiarise themselves with the response keys. In total, 102 pictures were presented twice in a random order (i.e. a total of 204 stimuli), representing the five emotion categories in ten different intensities expressed by two actors (one male, one female), and a neutral expression by the same two actors. Participants had to respond using labelled keys to indicate which of the five basic emotions was presented. A central fixation cross was displayed for 1000 ms between trials. This test resulted in a percentage accuracy for each facial expression as a function of intensity.

### Daily measures

In addition to the pre- and post-intervention assessments, participants were sent electronically a daily reminder to report their mood and stool characteristics using the online link provided. They could use the same link each day and submit their rating any time that day. Participants evaluated their mood each day by rating two statements on a scale from 0 to 100 using a sliding bar. Specifically, participants were asked to answer “How much positive feeling do you have today?” and “How much negative feeling do you have today?” An intercorrelation plot between day one mood reports and the pre-intervention questionnaire scores confirmed the expected correlations between them (Figure S2). The Bristol Stool Scale^63^ (BSS) was the third daily measure and was used to indicate which of the seven types of stool reflected their bowel movements in the past 24 hours.

### Statistical analyses

All statistical analyses were conducted using R 3.2.3 software^64^. For the questionnaire scores and dot-probe reaction times, the change following the intervention was calculated for each group. To test for a difference between the two groups, independent two-sample *t*-tests were performed where possible and Wilcoxon signed-rank tests were conducted when the assumptions of normality or homogeneity of variance were not met (determined using the Shapiro–Wilk test and Levene’s test respectively).

For the facial expression recognition task, a linear mixed-effects model was conducted with percent accuracy as the dependent variable, while the independent variables were emotion, intensity of emotion, group, session (i.e. pre- or post-intervention) and the interaction between group and session, along with participant ID number as a random effect.

Time series graphs were plotted for the three daily measures: positive mood, negative mood and Bristol Stool Scale. Linear mixed-effects models were constructed with the daily measure as the dependent variable, while the independent variables were group, time and the interaction between the two, along with participant ID number as a random effect. The results of these models were also confirmed using bootstrapping. An exploratory analysis was then conducted to determine whether any pre-intervention questionnaire scores predicted which individuals responded best to the probiotic treatment. For each participant, the Kendall Tau-b correlation coefficient of their change in negative mood over time was calculated and thus the more negative the correlation, the greater the decrease in negative mood. Pairwise Kendall Tau-b correlations between this coefficient of change in negative mood and the pre-intervention questionnaire scores were calculated and since this was an exploratory analysis, correlations where *P* ≤ 0.08 were plotted. A linear model to predict the change in negative mood was constructed and the inclusion of variables (i.e. pre-intervention questionnaire scores) was determined according to the Akaike Information Criterion (AIC), and the variance inflation factor (VIF) was calculated to confirm the absence of multicollinearity.

## Results

### Participant characteristics

In total, 88 healthy adult participants were tested, 44 in each group, with both groups showing similar characteristics (Table 1).

**Table 1:** Baseline participant characteristics

Characteristics	Probiotics	Placebo
Sample size	44 [15 M, 29 F]	44 [14 M, 30 F]
Age	22.3 (3.1)	22.3 (3.1)
BMI	23.4 (3.2)	23.6 (2.7)
PANAS - positive	27.8 (7.2)	29.5 (7.1)
PANAS - negative	14.5 (5.9)	15.9 (5.4)

Mean values are given with standard deviations in parentheses.

### Questionnaires

**Fig. 1:**
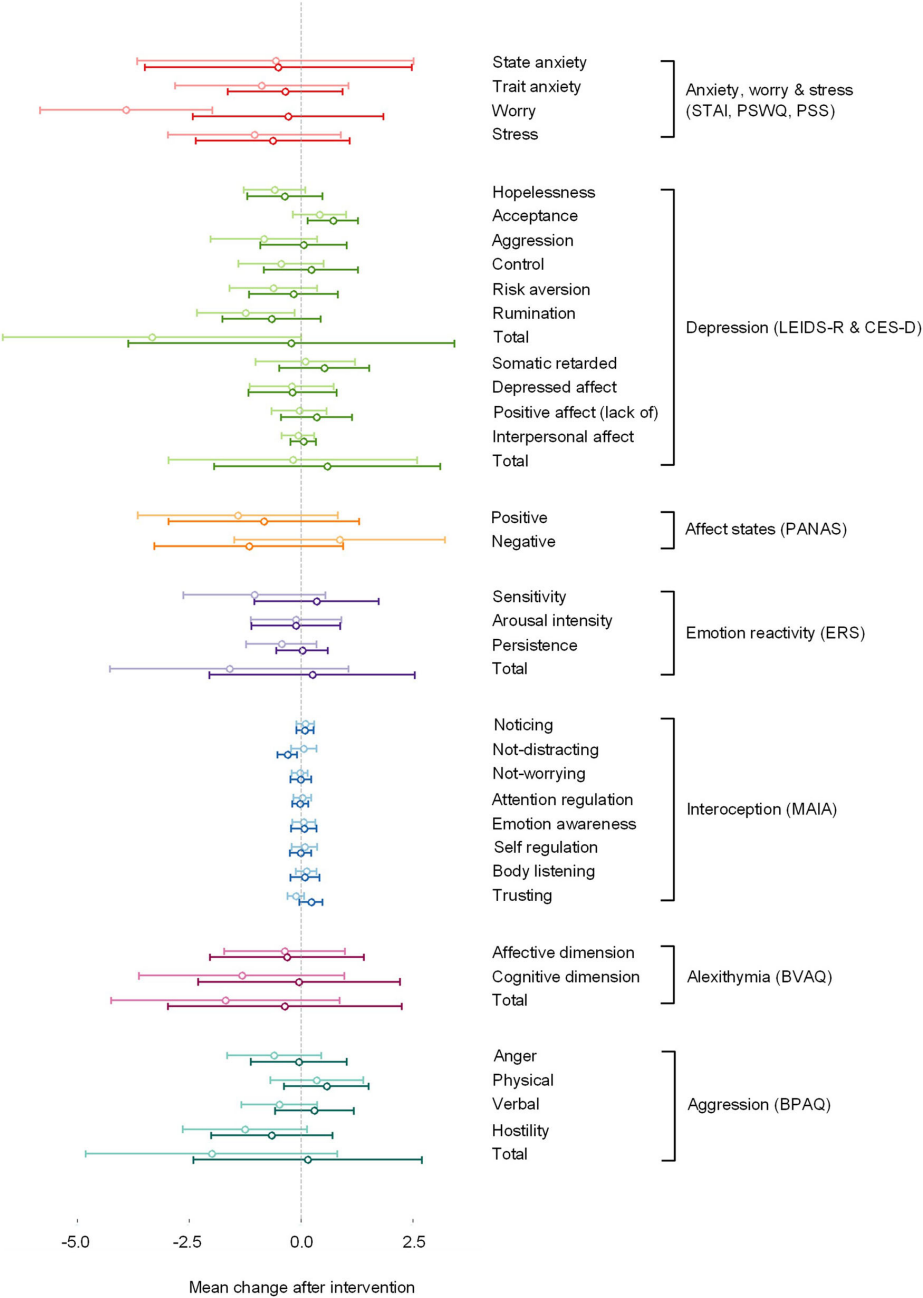
Change in questionnaire scores after intervention for the probiotic and placebo groups. The probiotic group (darker colour) is shown below the placebo group (lighter colour) and error bars depict 95% confidence interval of the mean change.

There was little evidence of differences between the probiotic and placebo groups in terms of the change in questionnaire scores following the intervention (Fig. 1, Table S1). The most notable difference was for the Penn State Worry Questionnaire; while the mean score for this questionnaire decreased for both groups following the intervention, only the placebo group demonstrated a significant reduction in worry (*P* = 0.018). Participants in the probiotic group scored lower on the not-distracting subscale of the MAIA following the intervention whereas there was little change in the placebo group. Thus people in the probiotic group showed a greater tendency to ignore or distract themselves from sensations of pain or discomfort following the intervention compared to the placebo group (*P* = 0.017). For the trusting subscale of the MAIA, measuring how much someone experiences their body as safe and trustworthy, there was a trend towards a difference between the two groups (*P* = 0.093) such that the mean change after treatment was greater in the probiotic group. However, given the number of questionnaires, the differences that were significant between the two groups would not be maintained following correction for multiple comparisons. In addition, there was no evidence of a treatment effect on either bowel complaints or frequency (Table S2) or the Bristol Stool Scale (Figure S1, Table S5).

### Emotional processing

For the dot-probe task, there was no evidence of a difference between the probiotic and placebo groups (*P* > 0.05) in terms of any change in bias towards the five emotions (Table S3). For the facial expression recognition task (Table S4), as expected the intensity of emotions predicted accuracy of recognition (*P* < 0.001), and the recognition accuracy also varied by the type of emotional expression (*P* < 0.05). There was a main effect of session (i.e. pre- or post-intervention), as participants were more accurate the second time around in the post-intervention testing session (*P* = 0.008), but there was no main effect of group. However, there was a marginally significant interaction between group and session (*P* < 0.05) such that the probiotic group showed improved accuracy in recognising facial expressions following the intervention.

### Daily reports

**Fig. 2:**
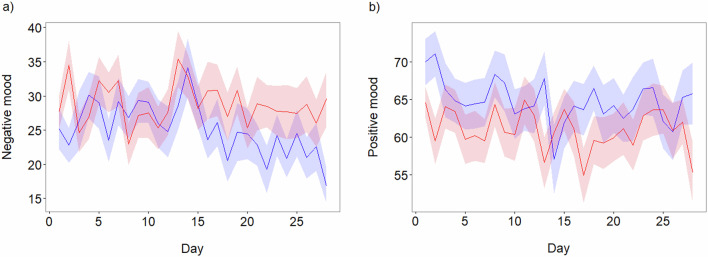
Change in daily mood scores during the 4-week intervention. Graphs depict the mean and standard error for **a** negative mood and **b** positive mood, with the probiotic group shown in blue and the placebo group in red.

The change in daily mood over time (Fig. 2), as measured by two visual analogue scales separately assessing positive and negative feelings, revealed a reduction in negative mood in the probiotic group, particularly following the two-week mark. Regression models (Table 2) found no main effect of group or time but the interaction between the two significantly predicted negative mood (*P* = 0.009), but not positive mood (*P* = 0.784) or stool consistency (*P* = 0.556, Figure S1, Table S5).

**Table 2:** Output from linear mixed-effects models predicting the change in daily mood scores during the 4-week intervention

Dependent variable	Model term	Coefficient	SE	Lower CL	Upper CL	*P* value
Negative mood	Group	−1.11	3.13	−7.23	5.02	0.724
	Time	−0.03	0.06	−0.15	0.09	0.601
	Group × Time	−0.22	0.08	−0.38	−0.06	0.009
Positive mood	Group	2.97	3.28	−3.45	9.39	0.367
	Time	−0.05	0.06	−0.16	0.06	0.382
	Group × Time	0.02	0.08	−0.13	0.18	0.784

Results are given with the estimate for the coefficient, standard error and 95% confidence limit (CL). Note that the placebo group was set as the reference level such that a negative coefficient indicates a lower mood score in the probiotic group.

### Predicting probiotic responders

**Fig. 3:**
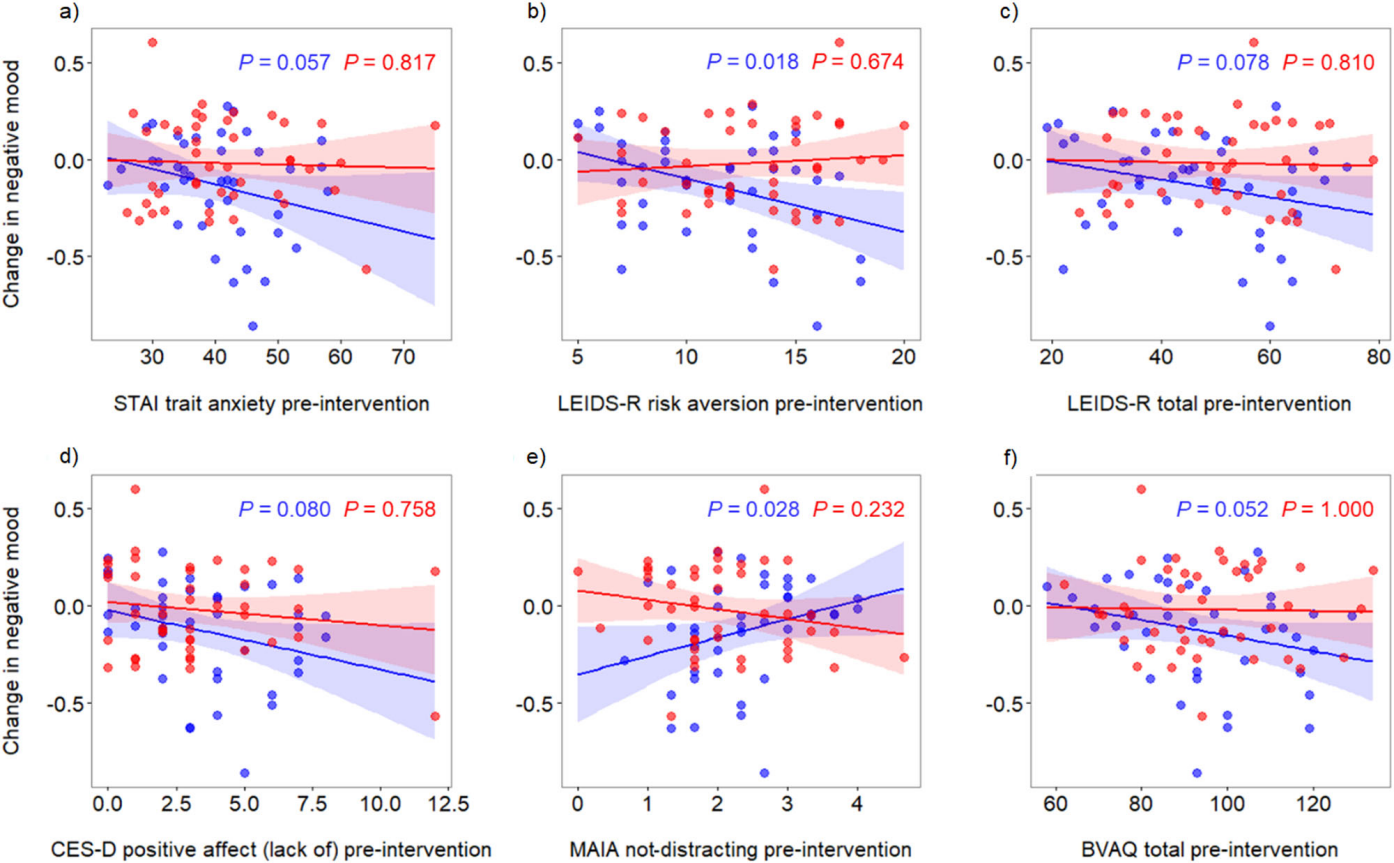
Correlation between pre-intervention questionnaire scores and the change in negative mood over time for the probiotic and placebo groups. Graphs show correlations where *P* ≤ 0.08 for the probiotic group, observed in relation to **a** STAI trait anxiety, **b** LEIDS-R risk aversion, **c** LEIDS-R total score, **d** CES-D positive affect (lack of), **e** MAIA not-distracting and **f** BVAQ total score. Shading depicts 95% confidence interval, with the probiotic group shown in blue and the placebo group in red.

In this exploratory analysis, six pre-intervention questionnaire scores showed some correlation (*P* ≤ 0.08) with the change in daily negative feelings in the probiotic group (Fig. 3). Notably, there was no evidence of such correlations in the placebo group (Fig. 3, Table S6).

A linear model to predict the change in negative mood (Table 3), constructed according to the Akaike Information Criterion (AIC), found that the main predictor of the change in negative mood was the LEIDS-R risk aversion subscale (*P* = 0.013).

**Table 3:** Output from linear model predicting the change in negative mood over time from pre-intervention questionnaire scores for the probiotic group

Model term	Coefficient	SE	Lower CL	Upper CL	*P* value
LEIDS-R risk aversion	−0.026	0.010	−0.047	−0.006	0.013
MAIA not-distracting	0.057	0.048	−0.040	0.154	0.241
BVAQ total	−0.003	0.002	−0.008	0.001	0.129

Results are given with the estimate for the coefficient, standard error and 95% confidence limit (CL).

## Discussion

In this comprehensive study, we find no evidence of a beneficial effect of probiotics using standard psychological questionnaires assessing constructs relating to emotion, yet we find a clear signal that probiotics improve negative mood in healthy volunteers based on daily reporting. Interestingly the results from the daily reporting reveal a decrease in negative mood but no change in positive mood. While it has previously been proposed that probiotics may function by reducing emotional reactivity to both positive and negative stimuli^65^, akin to the emotional blunting that is typically observed with antidepressants^66,67^, this does not align with our findings from the daily mood scores. If indeed it is the case that probiotics specifically reduce negative mood without simultaneously reducing positive mood, this could provide a notable benefit. However, since most probiotic studies investigate changes in depression, stress and anxiety, little is known about how probiotics impact positive affect, except one study (with a small sample size) that reported an increase in positive affect using the PANAS^40^, though no significant increase was observed in our study.

It is particularly striking that despite incorporating a range of questionnaires, the only differences following intervention were the placebo group scoring lower on the Penn State Worry Questionnaire and the probiotic group scoring lower on the not-distracting subscale of the MAIA, though given the number of questionnaires administered, definitive conclusions cannot be drawn from these findings. However, it is an interesting observation that for every questionnaire and subscale assessing emotion regulation (except the MAIA which measures interoception which is related to emotional states), the change following intervention is a lower score for the placebo group than the probiotic group. In fact, the only deviation is the PANAS, particularly negative affect where the probiotic group decreases following intervention and the placebo group increases slightly, though this difference is not significant. Since it is unlikely that this pattern shown by the majority of questionnaire scores is a chance occurrence, perhaps probiotics affect how individuals interpret their emotions in a way that influences the post-intervention assessment across questionnaires. While we cannot speculate too much regarding this pattern, it is a notable finding nonetheless that warrants further investigation.

In terms of the tests of emotional processing, there were no differences between the groups in performance on the dot-probe task. This is surprising as typically changes at the cognitive level are detected prior to subjective reporting. Indeed our previous research on other aspects of the microbiome–gut–brain axis, notably vagal signalling and antibiotic use, found changes in the dot-probe task, particularly bias towards sad faces^68,69^. For the facial expression recognition task, there was a marginally significant interaction effect of group and session, such that the probiotic group was more accurate at recognising emotions at the end of the intervention. In combination with the interesting pattern seen in the questionnaire scores, it is possible that probiotics may influence the ability to recognise emotions, both in oneself and in others. A research study using a different multispecies probiotic in subjects with untreated depression similarly found that it improved accuracy in the facial expression recognition task. In contrast, that study also found reduced vigilance towards both positive and negative stimuli in the dot-probe task in those taking probiotics, as well as a reduction in depression scores for the Patient Health Questionnaire-9^70^, perhaps because the participants recruited were suffering from depression, compared to our sample of healthy subjects.

For the field of the microbiome–gut–brain axis, our findings can help reconcile the inconclusive results of previous probiotic studies. We conclude that probiotics do have a beneficial effect on negative mood in a healthy population, but such an effect is not readily detected using the standard questionnaires that many studies rely on. Indeed, previous studies using healthy volunteers have reported a lack of effect in the main sample and only found an effect when limiting the sample to people reporting low mood or scoring high in diagnostic questionnaires prior to the intervention^42,71^. Based on our findings, we argue that while the psychotropic effects of probiotics may be more likely to be detected in people already suffering with psychological symptoms, this does not mean that they lack an effect in the general population. Our results indicate that daily mood measures provide a more sensitive method to assess changes in the participants’ emotional state following probiotics than standard questionnaires.

Given the rising levels of depression and prevalence of treatment-resistant depression, probiotic use in the general population may be particularly beneficial for certain individuals. Through exploratory analyses, we identify a psychological profile of individuals more likely to benefit from probiotics (Fig. 3), where risk aversion (as measured in the LEIDS-R) proved to be the most important predictor of improvement in negative mood. Research in clinical practice has concluded that the LEIDS-R is a valid measure of cognitive vulnerability to depression and, in particular, that rumination and risk avoidance might be the key driving factors^72^ which may help explain why most improvement in negative mood in our study was seen in those with higher baseline risk aversion. Early intervention with probiotics may provide a means by which individuals at risk of mental health conditions, such as those suffering low mood, may be able to reduce the chance of developing a clinical disorder, though more research would be needed to confirm such a use.

There are two ways in which the negative mood measure may have provided more insight than general standardised questionnaires: firstly, the openness and simplicity of the question and secondly, the temporal resolution enabled by the daily reports. While the PANAS negative affect score did reduce in the probiotic group following intervention compared to the placebo group where it increased slightly, this difference between the two groups was not significant. One reason for this may be because the questions assessing negative affect in the PANAS focus more on high arousal states such as distress and irritability, rather than aspects such as low mood and sadness. While asking participants to rate how positive or negative they are feeling on a scale from 0 to 100 without giving any further instruction might appear a crude measure, it also has the advantage that it does not attempt to dissect feelings and emotions into discrete categories. In recent decades psychology and psychiatry have turned away from subjective monitoring^73^, instead creating various constructs in an attempt to assess an individual’s emotional state more objectively. However, from a biological perspective we have little understanding of the mechanisms underpinning emotion regulation^74^ and it is likely there are shared processes involved in feelings of depression, anxiety and stress. Given the complexity of the brain, the subjective experience of mood offers a way to holistically evaluate how a person is feeling. Thus since the experience of emotion is very individual, questionnaires designed to objectively assess specific psychological constructs may fail to capture a general change in feeling, even though this is arguably more meaningful to the individual. Collecting data on participants’ mood may therefore be particularly useful with probiotic interventions since the exact psychological effects of probiotics are not well characterised or understood.

While these questionnaires assessing emotional regulation are validated with good consistency and their use is not limited to clinical populations, our results question the extent to which they may reliably detect less marked changes in a healthy population following an intervention. Furthermore, the majority of questionnaires assessing depressive symptoms do not focus on mood, despite depression being a mood disorder. Indeed negative mood may be more useful as it does not simply indicate depression but is also a factor that can detrimentally affect other elements such as sleep, cognitive function and suicide ideation^75^. Another important shortcoming of many of the questionnaires is that they add up items to derive a single score for a scale or subscale which requires the unlikely assumption that each contributes equally to the construct being measured^76^. Although self-monitoring can come with drawbacks, most notably it is considered more subjective than a questionnaire with specified questions and ratings, it also has the benefit of allowing the participant to report how they feel without being restricted within the context of a questionnaire. After all, we can ask participants every type of question, but perhaps in an attempt to delineate the complexity of the human brain and emotion, we have forgotten to ask the basics such as “How are you feeling today?” Arguably the answer to such a question is the most important and informative of all.

In addition, the daily collection of mood scores provides temporal resolution to understand the change in each group during the four-week intervention. Since participants were recruited in a staggered manner, spanning approximately one year, the patterns observed in their daily mood are not the result of general societal events. Our findings revealed that it may take on average around two weeks for probiotics to have an effect on reducing negative mood. One reason this is interesting is because it is roughly the same time it takes for any benefits of antidepressants to be felt by the patient. While antidepressants and probiotics seem rather different in their main mechanism of action, with the former designed to increase the availability of serotonin and the latter to introduce bacteria to the digestive system, there may be elements in common as both can have anti-inflammatory effects^14,77^ and may utilise signalling through the vagus nerve^32,78^. If there are some underlying similarities in the way they work, this may even help us understand why their effects appear to operate over comparable timescales.

Another advantage of using frequent reports from subjects is that it can help increase the validity of findings, providing more robust evidence of a change than just a difference in pre- and post-intervention scores which is more liable to chance. It also allows participants to score how they are feeling in their natural setting rather than completing questionnaires in the testing laboratory. In addition, post-intervention questionnaires can be prone to biases in recall and recency. While daily monitoring is a technique frequently used in the growing market of mental health-based apps, the majority of psychological research studies investigating the effects of an intervention rely only on pre- and post-measures. However, there is increasing interest in generating and analysing temporally rich datasets to evaluate the dynamics of emotion regulation in daily life via the use of ecological momentary assessments (EMAs)^79^. Currently, EMAs are largely being used in clinical psychology to understand the development of depression or suicidal behaviours^80,81^. Even single-item daily mood ratings via mobile phone have been found to strongly predict depression development^82^. In light of our findings, daily reporting offers potential benefits beyond clinical assessment as it can also provide more fine-scale understanding of the effects of an intervention over time.

The temporal insight from our findings also has implications for future studies on the effects of probiotics on human psychology. The majority of studies to date use an intervention period of three or four weeks. However, the divergence in negative mood seen in the latter stages of the four-week intervention indicates that potentially even a greater difference between the two groups may have been found if the study had continued for a longer duration. While we cannot extrapolate our results with certainty, this is an interesting avenue for future research, particularly since most people who take probiotics take them for an extended period of time, rather than just a few weeks.

In conclusion, we show via daily monitoring that probiotics can improve negative mood in healthy subjects. We also find that people with certain psychological traits, particularly those who are more risk averse, are more likely to show a greater reduction in negative mood when taking probiotics. Thus in the future probiotics may potentially be targeted to individuals to reduce the risk of clinical onset of mental health conditions. However, it is important to acknowledge that there remain unknowns regarding their precise mechanism of action and the array of effects probiotics can have on human physiology and psychology.

Additionally, our findings help reconcile the somewhat inconsistent results of human probiotic studies within the microbiome–gut–brain field to date. The lack of effects reported in numerous studies, particularly those from non-clinical samples, may derive from relying on pre- and post-intervention scores and using questionnaires that do not reliably capture the emotional change elicited by probiotics. Given our findings we propose that daily mood measures should be included in more studies, not just within the microbiome–gut–brain field but more widely when aiming to assess the effects of various interventions on mental health. In an attempt to assess mental health, we cannot lose sight of asking the obvious. Sometimes the most simple questions can reveal the most meaningful answers.

## Supplementary information

Supplementary material

## Data Availability

All stimuli, tasks, data and code needed to replicate the results can be accessed via this link: https://surfdrive.surf.nl/files/index.php/s/vWptthJOqQJHi3K. After publication, the contents of the above will be made available in the DataverseNL repository.
