# Supplementary material for: Probiotics reduce negative mood over time: the value of daily self-reports in detecting effects
Source: Npj Ment Health Res. 2025 Apr 9;4:10. doi: 10.1038/s44184-025-00123-z (PMC11982403; doi:10.1038/s44184-025-00123-z)
Supplement: Supplementary file 1 — Supplementary material [file 44184_2025_123_MOESM1_ESM.pdf]

Probiotics reduce negative mood over time: the value of daily self-reports in detecting effects

Katerina V.-A. Johnson & Laura Steenbergen

Supplementary Material

Explanation of questionnaires

The Bristol Stool Scale[1] (BSS) is a self-report scale designed to allow individuals to classify stool form. This scale requires participants to report the number of times they defecate each day, along with the form and consistency of their most recent stool, assessed on a seven-point pictorial scale. This scale was used since stool consistency has been shown to be strongly related to gut microbiome composition, richness and enterotypes[2,3] and stool frequency is also associated with gut microbiome composition[4,5]. In addition, the bowel complaints questionnaire assesses the frequency with which gastrointestinal symptoms are experienced and is based on the Gastro-Questionnaire developed to evaluate functional gastrointestinal disorders[6].

The State–Trait Anxiety Inventory[7] (STAI) is a commonly used measure to assess anxiety distinctive from depression. It assesses both state and trait anxiety by asking participants to rate statements on a scale from 1 (‘almost never’) to 4 (‘almost always’). Two scores are obtained, one for state anxiety and one for trait anxiety, each ranging between 20 and 80, with higher values indicating greater anxiety.

The Penn State Worry Questionnaire[8] (PSWQ) includes 16 items, with participants indicating the degree to which each characteristic best describes them on a scale from 1 (‘not at all typical of me’) to 5 (‘very typical of me’). Total scores can range from 16 to 80, with higher scores indicating greater worry.

The 10-item Perceived Stress Scale[9] (PSS) assesses the degree to which individuals perceive life as unpredictable, uncontrollable and overloading. Statements about feelings and thoughts during the last month are rated for frequency of occurrence from 0 (‘never’) to 4 (‘very often’). A total PSS score ranges between 0 and 40, with higher scores indicating higher levels of perceived stress.

The revised version of the Leiden Index of Depression Sensitivity[10] (LEIDS-R) assesses cognitive reactivity to sad mood, i.e. the extent to which an individual activates dysfunctional thought patterns. Participants are asked to imagine a day in which one feels down, but not depressed, and to rate 34 statements from 0 (‘not at all’) to 4 (‘completely applies to me’). This results in a total score ranging from 0 to 136, with higher scores indicating greater cognitive reactivity. Cognitive reactivity measured by this scale can be divided into six subscales that reflect more specific dysfunctional thought patterns: hopelessness, acceptance, aggression, control, risk aversion and rumination.

The Centre for Epidemiological Studies Depression Scale[11] (CES-D) includes 20 statements that are rated from 0 (‘rarely or none of the time’) to 3 (‘most or almost all of the time’). It identifies depression symptoms along four subscales: somatic-retarded activity, depressed affect, positive affect (lack of) and interpersonal affect. A total score ranges from 0 to 60, with higher scores indicating more depressive symptoms. The CES-D can also be used to identify individuals at risk for clinical depression[12].

The Positive and Negative Affect Schedule[13] (PANAS) includes 20 scales assessing 10 positive and 10 negative affect states. Participants are asked to rate, from 1 to 5, the extent to which they are experiencing each of the 20 presented affect states at that moment. A positive and a negative affect score, both ranging from 10 to 50, are obtained by adding the respective items.

The Emotion Reactivity Scale[14] (ERS) contains 21 self-report items intended to measure emotional reactivity, i.e. the tendency to experience emotional arousal, which can be divided into three components: emotion sensitivity, emotion intensity and emotion persistence. Statements are rated on a 5-point scale using 0 (‘not at all like me’) to 4 (‘completely like me’). Total scores range from 0 to 84, with higher scores indicating higher emotional reactivity.

The Multidimensional Assessment of Interoceptive Awareness[15] (MAIA) is a self-report measure for interoceptive body awareness along eight dimensions with 32 statements that are rated on a scale from 0 (‘never’) to 5 (‘always’). Respective items are averaged to assess interoception in terms of noticing bodily sensations; not distracting oneself from pain or discomfort; not worrying about feelings of pain or discomfort; being able to regulate attention for bodily sensations; being aware of emotional state and bodily sensations; being able to self-regulate distress by giving attention to bodily sensations; actively listening to one’s body to gain insight; and experiencing one’s body as safe.

The Bermond–Vorst Alexithymia Questionnaire[16] assesses difficulties in an individual’s ability to identify their own emotions (i.e. alexithymia). Participants are asked to rate 40 items on a scale from 1 (‘completely’) to 5 (‘not at all’), to indicate to what extent a certain statement applies to them. Higher scores represent more difficulty in identifying one’s own emotions. A total alexithymia score ranges from 40 to 200 and is obtained by adding items assessing both cognitive and affective understanding of one’s emotions.

The Buss–Perry Aggression Questionnaire[17] (BPAQ) contains 29 items used to calculate a total score ranging from 29 to 145. Participants indicate how applicable each item is on a scale from 1 (‘extremely uncharacteristic of me’) to 5 (‘extremely characteristic of me’). Aggression measured by this questionnaire can be divided into four subscales: anger, physical aggression, verbal aggression and hostility.

Pandemic impact

Since the active testing stage of the study took place between March 2019 and April 2020 (except no participants were involved over the Christmas period to exclude possible holiday effects[18]), some testing sessions were disrupted due to the global COVID-19 pandemic. Leiden University implemented safety measures entailing closure of all university buildings, including testing laboratories, as of 14 March 2020. No pre-intervention sessions were scheduled after this date and post-intervention sessions that had already been scheduled for after 14 March 2020 had to be

adjusted. On the day of the scheduled post-intervention session, exactly four weeks after starting the probiotics or placebo, participants were sent an e-mail providing access to the questionnaires. Participants were instructed to complete the questionnaires from their own device on that day and notify the experiment leader after completion. Consequently, for participants with this adjusted post-intervention session, the methods that required attendance at the testing laboratory could not be carried out. These adjusted measures were approved by the local ethics committee (Psychology Research Ethics Committee, Institute of Psychology, Leiden University) and affected 19 participants.

As a result, there were missing data for these participants for the tests of emotional processing and also the intended heart rate measurements (which were originally taken to assess heart rate variability[19] as this is considered a physiological measure of emotion regulation[20,21]). As well as the participants that could not complete the second session because of the start of the pandemic, the sample size for the heart rate analysis was further impacted due to some unreliable heart rate readings. After the heart rate data were recorded (using the wireless H7 Polar heart rate sensor[22] in conjunction with the Elite HRV application and processed with Kubios[23]), unusual signals were identified by the software as artefacts and resulted in data from an additional ten participants having to be excluded (see Table S7 for exclusion reasons). This, combined with the pandemic impact, substantially reduced the power of the subsequent heart rate analysis but for completeness the results are presented in Table S8, indicating no significant differences between the two groups in any of the measures.

Supplementary figures

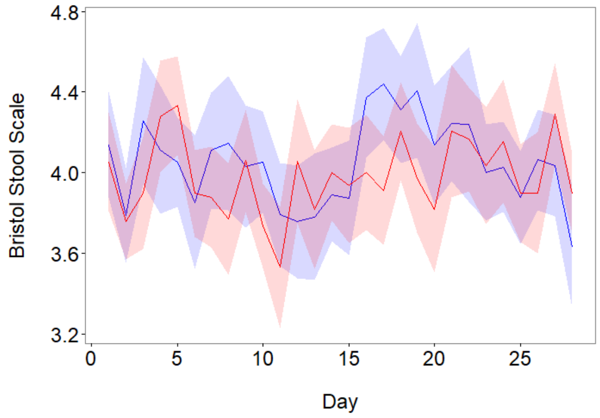

Figure S1. Change in daily Bristol Stool Scale during the four-week intervention. Graph depicts the mean and standard error, with the probiotic group shown in blue and the placebo group in red.

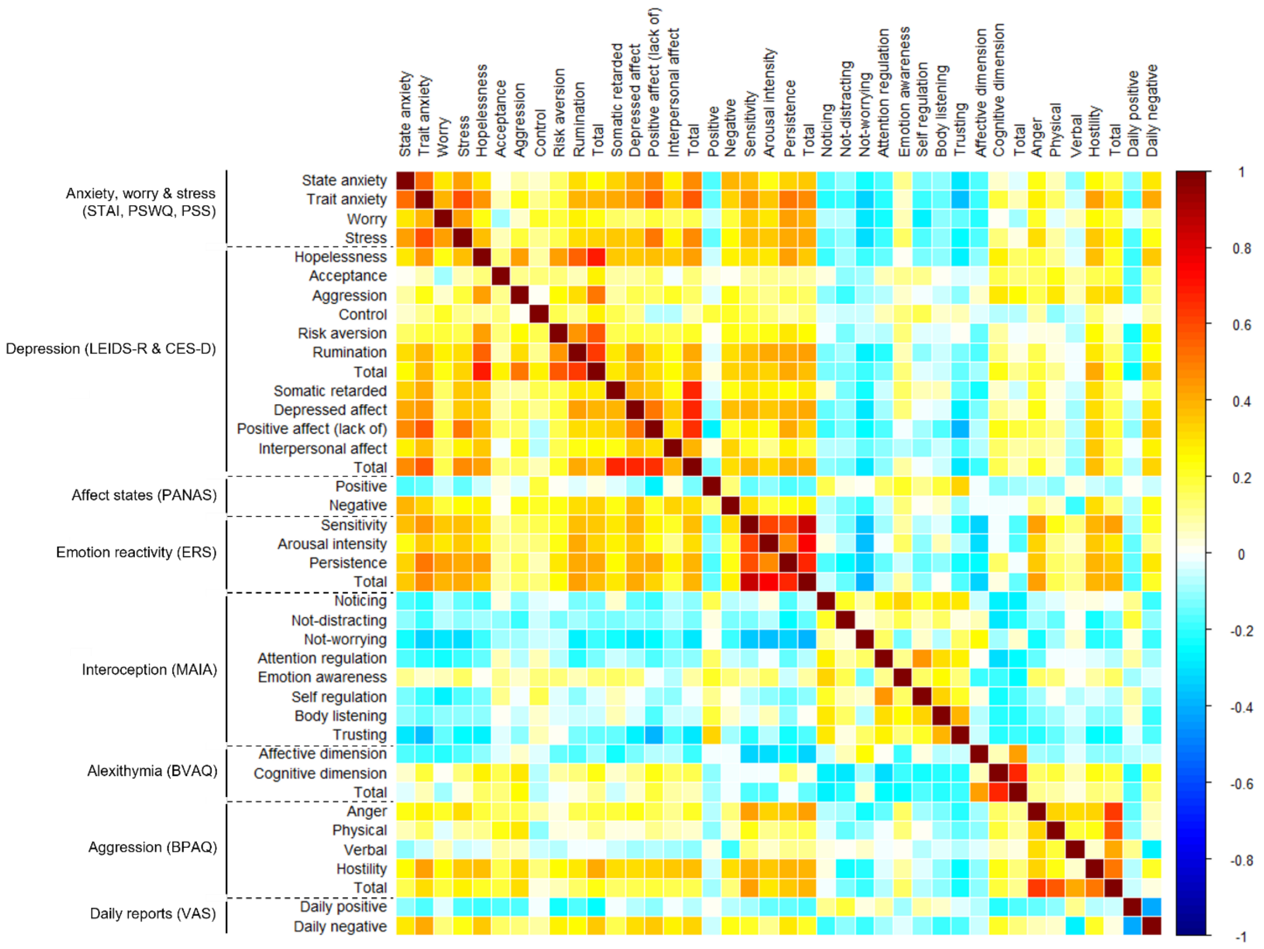

Figure S2. Intercorrelation plot for the pre-intervention questionnaire scores and day one mood reports. Plot depicts strength and direction of pairwise correlations, as measured by Kendall's Tau-b correlation coefficient.

Supplementary tables

Table S1. Mean questionnaire scores pre- and post-intervention and change in questionnaire scores after intervention for the probiotic and placebo groups. Table shows mean change in scores for questionnaires (and their subscales). Where the non-parametric Wilcoxon test† was conducted, the *Z* value is given instead of the *t* value.

Questionnaire (and subscales)		Pre-intervention		Post-intervention		Change		Statistics	
		Probiotics	Placebo	Probiotics	Placebo	Probiotics	Placebo	*t* or *Z* value	*P* value
State–Trait Anxiety Inventory (STAI)	State	35.59	37.21	35.08	36.64	-0.51	-0.57	-0.03	0.978
	Trait†	40.21	41.21	39.85	40.33	-0.36	-0.88	-0.19	0.846
Penn State Worry Questionnaire (PSWQ)†		48.34	51.47	48.05	47.56	-0.29	-3.91	-2.38	0.018
Perceived Stress Scale (PSS)		18.71	18.37	18.07	17.33	-0.63	-1.05	-0.32	0.748
Leiden Index of Depression Sensitivity – Revised (LEIDS-R)	Hopelessness†	4.37	5.31	4.00	4.71	-0.37	-0.60	-0.91	0.362
	Acceptance†	2.37	2.31	3.07	2.71	0.71	0.40	-0.70	0.486
	Aggression†	6.05	7.10	6.10	6.26	0.05	-0.83	-0.84	0.402
	Control	8.59	9.14	8.80	8.69	0.22	-0.45	-0.96	0.341
	Risk aversion†	11.12	12.88	10.95	12.26	-0.17	-0.62	-0.20	0.840
	Rumination†	12.63	14.29	11.98	13.05	-0.66	-1.24	-1.29	0.199
	Total†	45.12	51.02	44.90	47.69	-0.22	-3.33	-0.91	0.364
Centre for Epidemiologic Studies Depression Scale (CES-D)	Depressed affect†	3.10	3.02	2.90	2.81	-0.20	-0.21	-0.07	0.942
	Positive affect (lack of)†	3.56	3.30	3.90	3.26	0.34	-0.05	-0.91	0.365
	Somatic symptoms†	5.71	5.44	6.22	5.53	0.51	0.09	-0.54	0.590
	Interpersonal difficulties†	0.68	0.84	0.73	0.77	0.05	-0.07	-0.24	0.812
	Total†	14.10	13.72	14.68	13.53	0.59	-0.19	-0.21	0.837
Positive and Negative Affect Schedule (PANAS)	Positive	27.54	29.70	26.71	28.28	-0.83	-1.42	-0.39	0.701
	Negative†	14.39	16.02	13.22	16.88	-1.17	0.86	-0.11	0.914
Emotion Reactivity Scale (ERS)	Sensitivity	13.98	16.93	14.32	15.88	0.34	-1.05	-1.33	0.188
	Intensity	12.02	13.30	11.90	13.19	-0.12	-0.12	0.01	0.994
	Persistence†	7.07	7.91	7.10	7.47	0.02	-0.44	-0.74	0.460
	Total†	33.07	38.14	33.32	36.53	0.24	-1.60	-0.55	0.585
Multidimensional Assessment of Interoceptive Awareness (MAIA)	Noticing†	3.37	3.46	3.45	3.55	0.08	0.09	-0.79	0.429
	Not-distracting†	2.27	2.03	1.96	2.09	-0.31	0.05	-2.39	0.017
	Not-worrying	3.02	2.68	3.01	2.65	-0.01	-0.03	-0.16	0.877
	Attention regulation†	3.04	2.90	3.02	2.93	-0.02	0.03	-0.58	0.563
	Emotional awareness	3.38	3.48	3.43	3.53	0.06	0.06	-0.01	0.989
	Self-regulation†	2.88	2.76	2.87	2.84	-0.01	0.08	-0.48	0.631
	Body listening	2.27	2.21	2.35	2.33	0.08	0.12	0.18	0.861
	Trusting†	3.39	3.63	3.61	3.51	0.22	-0.12	-1.68	0.093
Bermond–Vorst Alexithymia Questionnaire (BVAQ)	Cognitive dimension	54.80	56.72	54.76	55.40	-0.05	-1.33	-0.80	0.425
	Affective dimension	40.83	38.93	40.51	38.56	-0.32	-0.37	-0.05	0.960
	Total	95.63	95.65	95.27	93.95	-0.37	-1.70	-0.74	0.464
Aggression Questionnaire (BPAQ)	Physical†	15.05	16.86	15.61	17.21	0.56	0.35	-0.45	0.652
	Verbal	14.98	15.26	15.27	14.77	0.29	-0.49	-1.29	0.201
	Anger	15.02	15.51	14.98	14.91	-0.05	-0.60	-0.75	0.457
	Hostility†	19.44	20.09	18.78	18.84	-0.66	-1.26	-1.08	0.281
	Total†	64.49	67.72	64.63	65.72	0.15	-2.00	-0.93	0.354

Table S2. Change in bowel complaints and frequency after intervention for the probiotic and placebo groups. Since the non-parametric Wilcoxon test was conducted, the *Z* value is given.

Bowel complaints & frequency	Probiotics	Placebo	*Z* value	*P* value
Loose stool	0.00	0.42	-1.92	0.055
Cramps	-0.02	-0.16	-0.64	0.522
Flatulence	0.32	0.21	-0.38	0.704
Constipation	0.00	0.09	-0.94	0.349
Bloating	0.20	-0.07	-1.85	0.064
Nausea	0.07	0.09	-0.90	0.366
Frequency	-0.05	0.02	-0.52	0.605

Table S3. Change in dot-probe task after intervention for the probiotic and placebo groups. Table shows mean change in reaction times (ms) for each emotional expression. Where the non-parametric Wilcoxon test† was conducted, the *Z* value is given instead of the *t* value.

Emotion	Probiotic	Placebo	*t* or *Z* value	*P* value
Sadness	9.21	3.36	-0.40	0.688
Fear	-2.02	3.29	0.37	0.715
Anger	-4.86	-1.90	0.19	0.851
Happiness†	1.18	2.35	-0.26	0.798
Surprise†	39.04	22.83	-0.52	0.600

Table S4. Output from linear mixed-effects model predicting percent accuracy in recognising emotional expressions. Results are given with the estimate for the coefficient, standard error and 95% confidence limit (CL). Note that the placebo group was set as the reference level for group (such that a positive coefficient indicates increased accuracy of recognition in the probiotic group) and anger was the reference level for emotion.

Model term	Coefficient	SE	Lower CL	Upper CL	*P* value
Group	-1.96	2.11	-6.08	2.16	0.355
Session (pre- or post-intervention)	2.34	0.88	0.62	4.05	0.008
Disgust	16.38	0.98	14.46	18.30	< 0.001
Fear	13.62	0.98	11.70	15.54	< 0.001
Happy	24.41	0.98	22.49	26.33	< 0.001
Sad	-0.55	0.98	-2.47	1.37	0.576
Emotion intensity	0.96	0.01	0.94	0.99	< 0.001
Group × Session	2.47	1.26	0.01	4.93	0.0499

Table S5. Output from linear mixed-effects model predicting the change in daily Bristol Stool Scale during the four-week intervention. Results are given with the estimate for the coefficient, standard error and 95% confidence limit (CL). Note that the placebo group was set as the reference level such that a negative coefficient indicates a lower score in the probiotic group.

Model term	Coefficient	SE	Lower CL	Upper CL	*P* value
Group	0.105	0.214	-0.312	0.523	0.622
Time	0.006	0.006	-0.005	0.016	0.318
Group × Time	-0.005	0.008	-0.020	0.011	0.556

Table S6. Correlation between pre-intervention questionnaire scores and the change in negative mood over time for the probiotic and placebo groups. Results are given for the pre-intervention scores that showed a correlation ($P \leq 0.08$) with the change in negative mood in the probiotic group.

Pre-intervention questionnaire	Group	Kendall’s Tau-b coeff	*P* value
STAI trait anxiety	Probiotics	-0.21	0.057
	Placebo	0.02	0.817
LEIDS-R risk aversion	Probiotics	-0.26	0.018
	Placebo	0.05	0.674
LEIDS-R total	Probiotics	-0.19	0.078
	Placebo	-0.03	0.810
CES-D positive affect (lack of)	Probiotics	-0.20	0.080
	Placebo	-0.03	0.758
MAIA not-distracting	Probiotics	0.25	0.028
	Placebo	-0.13	0.232
BVAQ total	Probiotics	-0.21	0.052
	Placebo	0.00	1.000

Table S7. Reasons for exclusion of participants based on heart rate data recorded. Unusual signals in the heart rate data are recorded by the software as artefacts and can occur for a number of reasons such as the participant moving (e.g. sneezing or coughing) or interference from another device. A small percentage of data rejected due to artefacts is acceptable but above 10% the data become too unreliable. While the aim was to collect heart rate data for 5 mins, this was not possible for every participant and a recording for a duration of less than 4 mins was excluded since it was not long enough to reliably extract heart rate variables. One recording also had to be removed because the RMSSD (root mean square of successive interbeat interval differences), a measure of heart rate variability, was considerably outside the normal range and thus treated as an anomaly.

Reason for exclusion	Recording (Participant ID)
> 10% artefacts	75 (140), 105 (156), 116 (162), 121 (164), 127 (168), 128 (168), 129 (169), 130 (169), 131 (170), 135 (172), 136 (172)
< 4 mins	64 (133)
Abnormally high RMSSD	31 (116)

Table S8. Change in heart rate variables after intervention for the probiotic and placebo groups. The heart rate variability measures assessed were RMSSD (root mean square of successive interbeat interval differences), SDNN (standard deviation of interbeat intervals) and pNN50 (percentage of successive interbeat intervals that differ by more than 50 ms). Where the non-parametric Wilcoxon test† was conducted, the *Z* value is given instead of the *t* value.

Heart rate & variability	Probiotics	Placebo	*t* or *Z* value	*P* value
Mean heart rate (bpm)	2.08	1.41	-0.23	0.820
Min heart rate (bpm)	0.93	2.01	0.42	0.674
Max heart rate (bpm)†	0.41	1.21	-0.81	0.420
RMSSD (ms)†	-4.06	-8.99	-0.86	0.393
SDNN (ms)†	-4.57	-8.39	-0.74	0.459
pNN50	-3.03	-5.59	-0.54	0.595

Supplementary references

1. Lewis, S. J. & Heaton, K. W. Stool form scale as a useful guide to intestinal transit time. *Scand. J. Gastroenterol.* **32**, 920–924 (1997).
2. Vandeputte, D. *et al.* Stool consistency is strongly associated with gut microbiota richness and composition, enterotypes and bacterial growth rates. *Gut* **65**, 57–62 (2016).
3. Tigchelaar, E. F. *et al.* Gut microbiota composition associated with stool consistency. *Gut* **65**, 540–542 (2016).
4. Kwon, H. J. *et al.* Is stool frequency associated with the richness and community composition of gut microbiota? *Intest. Res.* **17**, 419–426 (2019).
5. Hadizadeh, F. *et al.* Stool frequency is associated with gut microbiota composition. *Gut* **66**, 559–560 (2017).
6. Leibbrand, R., Cuntz, U. & Hiller, W. Assessment of functional gastrointestinal disorders using the Gastro-Questionnaire. *Int. J. Behav. Med.* **9**, 155–172 (2002).
7. Spielberger, C. D., Gorsuch, R. L., Lushene, R., Vagg, P. R. & Jacobs, G. A. *Manual for the State-Trait Anxiety Inventory*. (Consulting Psychologists Press, 1983).
8. Meyer, T. J., Miller, M. L., Metzger, R. L. & Borkovec, T. D. Development and validation of the Penn State Worry Questionnaire. *Behav. Res. Ther.* **28**, 487–495 (1990).
9. Cohen, S., Kamarck, T. & Mermelstein, R. A global measure of perceived stress. *J Heal. Soc Behav.* **24**, 385–396 (1983).
10. Van der Does, A. & Williams, J. Leiden Index of Depression Sensitivity – Revised (LEIDS-R). *Leiden University* (2003). Available at: http://www.dousa.nl/publications_ depression.htm#LEIDS.
11. Radloff, L. The CES-D scale: a self-report depression scale for research in the general population. *Appl. Psychol. Meas.* **1**, 385–401 (1977).
12. Lewinsohn, P. M., Seeley, J. R., Roberts, R. E. & Allen, N. B. Center for Epidemiologic Studies Depression Scale (CES-D) as a screening instrument for depression among community-residing older adults. *Psychol. Aging* **12**, 277–287 (1997).
13. Watson, D., Clark, L. A. & Tellegen, A. Development and validation of brief measures of positive and negative affect: the PANAS scales. *Journal of Personality and Social Psychology* **54**, 1063–1070 (1988).
14. Nock, M. K., Wedig, M. M., Holmberg, E. B. & Hooley, J. M. The Emotion Reactivity Scale: development, evaluation, and relation to self-injurious thoughts and behaviors. *Behav. Ther.* **39**, 107–116 (2008).
15. Mehling, W. E. *et al.* The Multidimensional Assessment of Interoceptive Awareness (MAIA). *PLoS One* **7**, e48230 (2012).
16. Vorst, H. C. M. & Bermond, B. Validity and reliability of the Bermond–Vorst Alexithymia Questionnaire.

Pers. Individ. Dif. **30**, 413–434 (2001).

17. Buss, A. H. & Perry, M. The aggression questionnaire. *J. Pers. Soc. Psychol.* **63**, 452–459 (1992).
18. de Clercq, N. C. *et al.* The effect of having Christmas dinner with in-laws on gut microbiota composition. *Hum. Microbiome J.* **13**, 10058 (2019).
19. Task Force of The European Society of Cardiology and The North American Society of Pacing and Electrophysiology. Heart rate variability: standards of measurement, physiological interpretation, and clinical use. *Eur. Heart J.* **17**, 354–381 (1996).
20. Williams, D. W. P. *et al.* Resting heart rate variability predicts self-reported difficulties in emotion regulation: a focus on different facets of emotion regulation. *Front. Psychol.* **6**, 1–8 (2015).
21. Mather, M. & Thayer, T. How heart rate variability affects emotion regulation brain networks. *Curr. Opin. Behav. Sci.* **19**, 98–104 (2019).
22. Weippert, M. *et al.* Comparison of three mobile devices for measuring R-R intervals and heart rate variability: Polar S810i, Suunto t6 and an ambulatory ECG system. *Eur. J. Appl. Physiol.* **109**, 779–786 (2010).
23. Tarvainen, M. P., Niskanen, J. P., Lipponen, J. A., Ranta-aho, P. O. & Karjalainen, P. A. Kubios HRV – heart rate variability analysis software. *Comput. Methods Programs Biomed.* **113**, 210–220 (2014).
